# Increased Risk of Early-Onset Endometrial Cancer in Women Aged 20–39 Years with Non-Alcoholic Fatty Liver Disease: A Nationwide Cohort Study

**DOI:** 10.3390/cancers17081322

**Published:** 2025-04-14

**Authors:** Joo-Hyun Park, Jung Yong Hong, Kyungdo Han, Wonseok Kang, Jay J. Shen

**Affiliations:** 1 Department of Family Medicine, Korea University Ansan Hospital, Korea University College of Medicine, Ansan 15355, Republic of Korea; joohyun_park@korea.ac.kr; 2 Department of Healthcare Administration and Policy, School of Public Health, University of Nevada Las Vegas, Las Vegas, NV 89154, USA; jay.shen@unlv.edu; 3 Division of Hematology-Oncology, Department of Medicine, Samsung Medical Center, Sungkyunkwan University School of Medicine, Seoul 06351, Republic of Korea; 4 Department of Clinical Research Design and Evaluation, Samsung Advanced Institute for Health Science and Technology (SAIHST), Sungkyunkwan University, Seoul 03181, Republic of Korea; 5 Department of Statistics and Actuarial Science, Soongsil University, Seoul 06978, Republic of Korea; 6 Division of Gastroenterology, Department of Medicine, Samsung Medical Center, Sungkyunkwan University School of Medicine, Seoul 06351, Republic of Korea

**Keywords:** endometrial neoplasms, non-alcoholic fatty liver disease, young adult, obesity, risk factors

## Abstract

**Background/Objectives**: Given the rising incidence of early-onset endometrial cancer diagnosed before the age of 50 years, this study examined whether non-alcoholic fatty liver disease (NAFLD) served as an independent risk factor for early-onset endometrial cancer, irrespective of obesity status. **Methods**: This nationwide cohort study included 2,311,949 Korean women aged 20–39 years who underwent health screenings from 2009 to 2012. NAFLD severity was classified based on the fatty liver index: none (<30), moderate (30–59), and severe (≥60). Multivariable-adjusted Cox proportional hazards models were applied to estimate adjusted hazard ratios (aHRs) for early-onset endometrial cancer. **Results**: During a median follow-up of 7.6 years, 1289 women developed early-onset endometrial cancer. The cumulative incidence of early-onset endometrial cancer was significantly higher in women with NAFLD than in those without (log-rank *p* < 0.001). A dose-dependent association was observed, with increased risk corresponding to greater NAFLD severity (aHR [95% confidence interval (CI)]: moderate NAFLD, 2.38 [1.99–2.85]; severe NAFLD, 5.39 [4.44–6.53]; *p* for trend < 0.01). Compared with non-obese women without NAFLD, the aHRs for early-onset endometrial cancer were 2.53 [2.11–3.05] in non-obese women with NAFLD, 1.66 [1.10–2.52] in obese women without NAFLD, and 4.30 [3.60–5.13] in obese women with NAFLD (synergy index = 1.50, *p* < 0.01). **Conclusions**: NAFLD was independently associated with increased risk of early-onset endometrial cancer in both non-obese and obese women. Furthermore, young women with both NAFLD and obesity exhibited a synergistically elevated risk. Early identification and management of NAFLD may help mitigate the rapidly growing burden of early-onset endometrial cancer.

## Simple Summary

The incidence of endometrial cancer in young women has been increasing, necessitating the identification of novel risk factors beyond obesity. This study investigated whether non-alcoholic fatty liver disease (NAFLD), a common metabolic condition, is independently associated with the risk of early-onset endometrial cancer. Utilizing nationwide health screening data from over two million Korean women aged 20 to 39 years, the analysis demonstrated that NAFLD was significantly associated with an elevated risk of endometrial cancer, regardless of obesity status. Moreover, the combination of NAFLD and obesity was linked to a synergistically increased risk. These findings highlight the potential value of early detection and management of NAFLD in mitigating the growing burden of endometrial cancer among young women.

## 1. Introduction

Endometrial cancer is increasing in both incidence and associated mortality worldwide [1,2,3]. Although the incidence of endometrial cancer has increased across all age groups, the rate of early-onset cases has risen more rapidly among reproductive-aged women, with an annual increase of 2.2%, twice the rate seen in women over 50 [1,3,4,5,6,7]. The majority of early-onset endometrial cancer cases are endometrioid adenocarcinomas and are associated with excessive estrogen exposure [8]. The most common causes of inherited early-onset endometrial cancer include Lynch syndrome and Cowden syndrome; however, these genetic conditions cannot explain the rapidly increasing incidence of early-onset endometrial cancer. Hereditary cases account for approximately 6–8% of all early-onset endometrial cancer cases, and their prevalence has remained relatively stable over the past few decades [7,8].

Unlike elderly women, early detection is more challenging in young women because vaginal bleeding, an early symptom of endometrial cancer, can be mistaken for benign conditions such as irregular menstruation and the perimenopausal transition [7,9]. Moreover, despite treatment, women with early-onset endometrial cancer may experience long-term complications, including impaired reproductive function, premature menopause, and psychosocial dysfunction [10,11,12,13,14]. Thus, the prevention of early-onset endometrial cancer through the mitigation of its risk factors is crucial.

Obesity is a well-established risk factor for early-onset endometrial cancer, possibly owing to mechanisms involving chronic inflammation, insulin resistance, and increased estrogen levels [15,16]. Growing evidence suggests that non-alcoholic fatty liver disease (NAFLD), a systemic metabolic disorder, is associated with chronic inflammation and endocrine dysfunction, such as systemic insulin resistance and hyperestrogenism, and serves as a risk factor for several types of cancer [17,18,19,20,21,22,23,24]. However, the association between NAFLD and the risk of early-onset endometrial cancer has not been comprehensively investigated. Few small-scale studies have suggested a positive [23,24] or no significant association [25,26,27,28] between NAFLD and the risk of endometrial cancer among middle- and older-aged women (median age, 53–54 years; ≤76 endometrial cancer cases in the NAFLD group).

We therefore conducted a large-scale, population-based, nationwide cohort study of over 2.3 million young women aged 20–39 years to investigate the association between NAFLD and the risk of early-onset endometrial cancer, independent of obesity status. We also examined the individual and combined associations of NAFLD and obesity with the risk of early-onset endometrial cancer.

## 2. Methods

### 2.1. Data Source

For this nationwide, population-based cohort study, data from the Korean National Health Insurance Service (KNHIS) database, which is linked to the National Health Screening Program, were analyzed. The KNHIS is a universal, government-implemented national health insurance program that provides coverage for over 97% of the population. The KNHIS database includes demographic information, diagnoses coded according to the International Classification of Diseases, 10th Revision, Clinical Modification (ICD-10-CM), prescriptions, procedures, and hospital utilization data. The National Health Screening Program comprises anthropometric measurements, blood and urine tests, and a self-administered questionnaire on medical history and lifestyle factors. Mortality information was obtained from the National Death Registry.

This study was approved by the Institutional Review Board (IRB) of Samsung Medical Center (IRB No. SMC2021-05-066). The requirement for written informed consent was waived as the dataset was anonymized to protect personal information. This study adhered to the principles outlined in the Declaration of Helsinki.

### 2.2. Study Population

**Figure 1:**
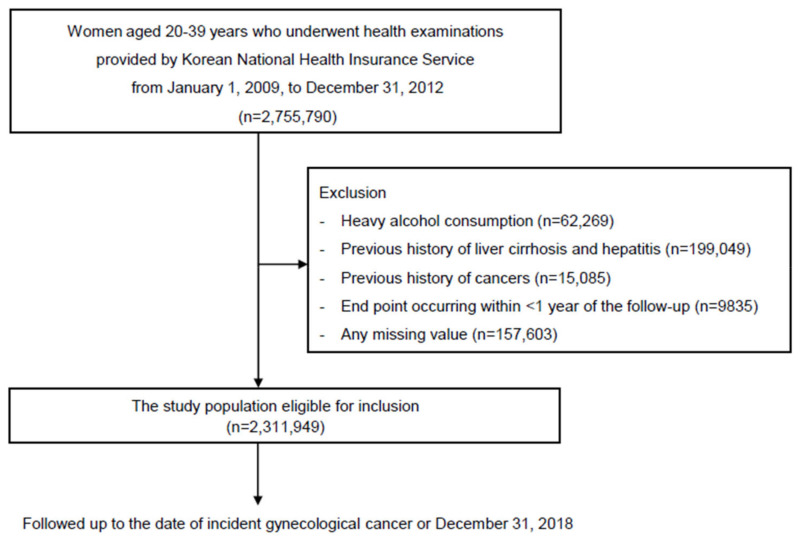
Study population selection flow chart.

The selection process for the study population is illustrated in Figure 1. A total of 2,755,790 women aged 20–39 years who participated in the National Health Screening Program from 1 January 2009 to 31 December 2012 were included. Exclusion criteria were as follows: participants with a preexisting diagnosis of cancer at baseline (*n* = 15,085); those who reported heavy alcohol consumption (≥20 g of alcohol per day, *n* = 62,269); individuals diagnosed with viral hepatitis or alcoholic liver cirrhosis (ICD-10-CM codes B15–B19 and K70.3, respectively) (*n* = 199,049); and those with missing data (*n* = 157,603). To reduce potential bias, individuals who developed cancer or died within the first year of follow-up (lag period) were also excluded (*n* = 9835). Thus, a total of 2,311,949 women were included and followed from baseline until the earliest occurrence of incident early-onset endometrial cancer, death, or 31 December 2018.

### 2.3. Anthropometrics and Laboratory Measurements

During the National Health Screening Program, healthcare professionals assessed the following parameters. Participants’ height, weight, and waist circumference were measured while they wore lightweight clothing. Body mass index (BMI) was calculated as weight (kg) divided by the square of height (m^2^). Systolic and diastolic blood pressures were measured while participants were seated after at least 5 min of rest.

Following an overnight fast of at least 8 h, blood samples were collected to measure glucose, total cholesterol, triglycerides, low-density lipoprotein cholesterol, high-density lipoprotein cholesterol, creatinine, aspartate aminotransferase, alanine aminotransferase, and γ-glutamyl transferase (GGT) levels. Laboratory testing for hepatitis A, B, and other hepatotropic viruses was not performed; however, information on these infections was obtained based on corresponding diagnostic codes. The estimated glomerular filtration rate (eGFR) was calculated using the Modification of Diet in Renal Disease (MDRD) study equation [29].

### 2.4. Definition of NAFLD

NAFLD was defined by the presence of hepatic steatosis in the absence of viral hepatitis or heavy alcohol consumption (≥20 g/day in women) [30]. In this study, which included 2.3 million young women from the general population, hepatic steatosis was defined using the fatty liver index [31]. The fatty liver index is an extensively validated predictive model with adequate accuracy for detecting fatty liver [32,33,34]. The index was calculated using the following equation [30,31]:

(e^0.953 × Ln (triglycerides) + 0.139 × BMI + 0.718 × Ln (GGT) + 0.053 × waist circumference ± 15.745^)/(1 + e^0.953 × Ln (triglycerides) + 0.139 × BMI + 0.718 × Ln (GGT) + 0.053 × waist circumference ± 15.745^) × 100.

The optimal cutoff value of the fatty liver index for detecting ultrasonography-diagnosed fatty liver in the general Korean population has been previously validated as ≥30 [18,32,33,34,35,36]. To investigate the dose-dependent association of hepatic steatosis, participants were classified into three groups based on their NAFLD status, as follows: none (fatty liver index < 30), moderate (fatty liver index 30–59), and severe (fatty liver index ≥ 60) [32,33,35]. The fatty liver index has been widely used in numerous population-based studies as a reliable surrogate marker of hepatic steatosis [18,33,34,35,36,37].

### 2.5. Study Outcome

The primary outcome of this study was newly diagnosed early-onset endometrial cancer, defined as a diagnosis occurring in women aged 20–49 years. Early-onset endometrial cancer was identified using ICD-10-CM codes C54, recorded during hospital admission, along with the special reimbursement code for cancer (V193). The KNHIS special copayment reduction program for cancer significantly alleviates the financial burden on cancer patients. Enrollment in this program requires physician certification, ensuring the reliability of the cancer diagnosis variable, which has been validated in previous studies [38,39].

### 2.6. Clinical Variables

Information on alcohol consumption, smoking status, and physical activity was obtained through a standardized self-assessment questionnaire. Light-to-moderate alcohol consumption in women was defined as <20 g of alcohol per day. Participants were categorized as never, former, or current smokers based on their smoking history. Regular physical activity was defined as ≥20 min of vigorous-intensity physical activity at least three times per week or ≥30 min of moderate-intensity physical activity at least five times per week. Low-income status was defined as belonging to the lowest income quintile or qualifying for medical aid benefits.

Obesity was defined as a BMI ≥ 25 kg/m^2^, and abdominal obesity was defined as a waist circumference ≥ 85 cm for women, based on Korean-specific criteria [40,41]. Diabetes was defined as a fasting glucose level ≥ 126 mg/dL or at least one insurance claim per year for an antidiabetic medication prescription (oral and/or injectable), classified under ICD-10-CM codes E11–E14. Dyslipidemia was defined as a total cholesterol level ≥240 mg/dL or at least one claim per year for lipid-lowering medication under ICD-10-CM code E78. Hypertension was defined as a systolic blood pressure ≥ 140 mmHg, a diastolic blood pressure ≥ 90 mmHg, or at least one annual claim for an antihypertensive prescription under ICD-10-CM codes I10–I13 or I15. Chronic kidney disease (CKD) was defined as an eGFR < 60 mL/min/1.73 m^2^. Pelvic inflammatory disease and polycystic ovary syndrome were identified using ICD-10-CM codes N73.9 and E28.2, respectively.

### 2.7. Statistical Analysis

The incidence rates of early-onset endometrial cancer were calculated as the number of incident cases divided by the total follow-up duration and expressed per 100,000 person-years. The log-rank test was used to compare Kaplan–Meier curves depicting the cumulative incidence of early-onset endometrial cancer in women with and without NAFLD.

Cox proportional hazards regression models were used to estimate hazard ratios (HRs) and 95% confidence intervals (CIs). The multivariate models were adjusted for basic demographic factors (age), personal characteristics (smoking status, alcohol consumption, physical activity, and income level), and comorbidities (obesity, diabetes, pelvic inflammatory disease, and polycystic ovary syndrome). Three models were constructed: Model 1 was unadjusted; Model 2 was adjusted for age; and Model 3 was further adjusted for smoking status, alcohol consumption, physical activity, income level, obesity, diabetes, pelvic inflammatory disease, and polycystic ovary syndrome. The proportional hazards assumption was evaluated using Schoenfeld residuals and log-log plots. The variance inflation factor (VIF) was computed to assess multicollinearity, with a VIF <5 indicating no significant collinearity. To examine the trend in risk estimates across categories, we calculated the *p* for trend using logistic regression.

Additionally, we examined the combined effect of NAFLD and obesity on the risk of early-onset endometrial cancer. The synergy index was used to determine whether the combined effect of these two risk factors exceeded their individual effects. The synergy index was calculated as follows: S = [HR_A+B+_ − 1]/(HR_A+B−_ − 1) + (HR_A−B+_ − 1), where HR_a+_B_+_ represents the HR for the combined effect of both risk factors, and HR_a+_B_−_ and HR_a−_B_+_ represent the HRs of each individual risk factor in the absence of the other. [42] An S value of 1 indicates no interaction (exact additivity), S < 1 suggests a negative interaction (less than additivity), and S > 1 indicates a positive interaction (greater than additivity). Statistical significance was defined as a two-sided *p*-value < 0.05. All analyses were conducted using SAS software (version 9.4; SAS Institute, Cary, NC, USA).

## 3. Results

### 3.1. Baseline Characteristics of the Study Population

Table 1 presents the baseline characteristics of the study population. Among the 2,311,949 women (mean [standard deviation] age: 29.7 [5.2] years), 1289 cases of early-onset endometrial cancer were diagnosed during 16,863,075 person-years of follow-up. Women who developed early-onset endometrial cancer were older than those who did not (*p* < 0.01). Additionally, women with early-onset endometrial cancer were more likely to have diabetes, hypertension, dyslipidemia, and obesity (all *p* < 0.01).

### 3.2. Risk of Young-Onset Endometrial Cancer According to NAFLD Status

**Figure 2:**
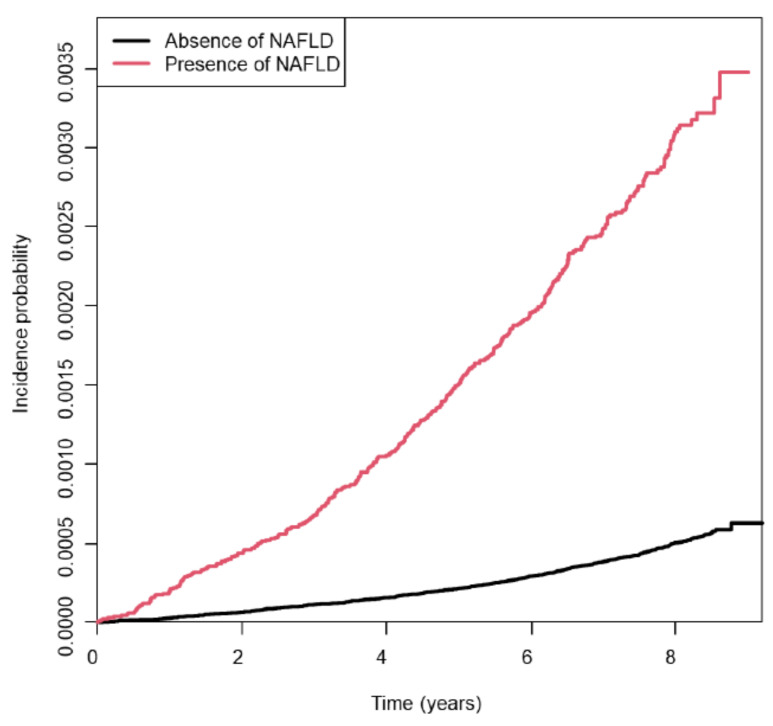
Kaplan–Meier curves of incidence probability of early-onset endometrial cancer according to NAFLD status (log-rank *p* < 0.01). Abbreviations: NAFLD, non-alcoholic fatty liver disease.

The Kaplan–Meier curves demonstrated that the cumulative incidence rates of early-onset endometrial cancer were consistently higher in women with NAFLD than in those without NAFLD throughout the follow-up period (log-rank *p* < 0.001) (Figure 2). Table 2 shows that the incidence of early-onset endometrial cancer was significantly higher among women with NAFLD (36.5 per 100,000 person-years) than among those without NAFLD (5.9 per 100,000 person-years) (*p* < 0.01).

NAFLD was significantly associated with increased risk of early-onset endometrial cancer after adjusting for potential confounders (Model 3: HR [95% CI]: 3.19 [2.76–3.67]). A dose-dependent relationship was observed, wherein the risk of early-onset endometrial cancer increased progressively with NAFLD severity (Model 3: HR [95% CI]: moderate NAFLD, 2.38 [1.99–2.85]; severe NAFLD, 5.39 [4.44–6.53]; *p* for trend < 0.01). The collinearity analysis revealed no evidence of collinearity among the variables.

### 3.3. Risk of Young-Onset Endometrial Cancer According to the Combination of NAFLD and Obesity

**Figure 3:**
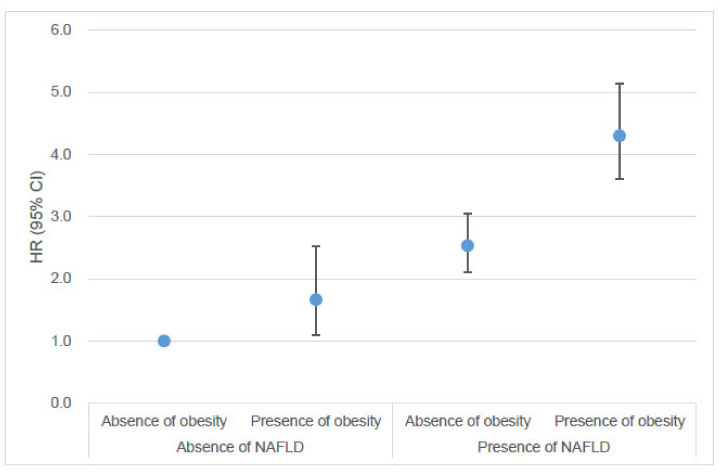
Risk of early-onset endometrial cancer according to the combination of NAFLD and obesity. Hazard ratios and 95% confidence intervals were adjusted for age, smoking status, alcohol consumption, regular physical activity, income status, obesity, diabetes, pelvic inflammatory disease, and polycystic ovary syndrome. Abbreviations: CI, confidence interval; HR, hazard ratio; NAFLD, non-alcoholic fatty liver disease.

Figure 3 illustrates the individual and combined effects of NAFLD and obesity on the risk of early-onset endometrial cancer. Compared to non-obese women without NAFLD, obese women without NAFLD had an increased risk of early-onset endometrial cancer, while non-obese women with NAFLD exhibited a further increased risk (Model 3: HR [95% CI]: 1.66 [1.10–2.52] and 2.53 [2.11–3.05], respectively).

The highest risk of early-onset endometrial cancer was observed in obese women with NAFLD, who had a 4.30-fold increased risk compared to non-obese women without NAFLD (Model 3: HR [95% CI]: 4.30 [3.60–5.13]). The combination of NAFLD and obesity was associated with a synergistically increased risk of early-onset endometrial cancer (synergy index = 1.50, *p* < 0.01).

## 4. Discussion

In this nationwide, population-based cohort study of more than 2.3 million young women, we established that NAFLD was independently associated with an increased risk of early-onset endometrial cancer, irrespective of obesity status. A dose-dependent relationship was observed, wherein the risk of early-onset endometrial cancer increased progressively with NAFLD severity. Moreover, young women with both NAFLD and obesity exhibited a synergistically elevated risk of early-onset endometrial cancer.

Previous small-scale studies have reported inconsistent findings, with both positive [23,24] and no significant associations [25,26,27,28] between NAFLD and endometrial cancer risk among middle-aged and older women. A positive association has been reported in cohort studies conducted in the United States (incidence rate ratio [95% CI]: 2.3 [1.4–4.1]; median age: 54 years; 76 cases in the NAFLD group) and Sweden (HR [95% CI]: 1.78 [1.18–2.68]; median age: 53 years; 25 cases in the NAFLD group) [23,24]. In addition, a meta-analysis of 10 observational studies revealed that NAFLD was associated with a higher risk of gynecological (uterine and ovarian) cancers (pooled random-effects HR [ 95% CI]: 1.62 [1.13–2.32]; mean age: 51 years; 558 gynecological cancer cases) [43]. Their findings were consistent with the results obtained in the present study.

Conversely, other small-scale studies investigating the association between NAFLD and the risk of gynecological cancers (uterine, cervical, and ovarian) reported no significant association [25,26,27,28]. The difference from our findings may be due to their smaller sample sizes and the inclusion of multiple gynecological cancers (uterine, cervical, and ovarian) as outcomes, rather than uterine cancer alone. Moreover, these previous studies employed various diagnostic methods for NAFLD, including ultrasonography [25,28] and ICD-9/10-CM codes [23,24,26]. Our study utilized the fatty liver index, an objective and validated tool with high feasibility for large-scale population studies, which has been widely used in epidemiological research, and demonstrated a dose–response relationship between hepatic steatosis severity and the risk of early-onset endometrial cancer. To the best of our knowledge, this is the first and largest study to demonstrate the association between NAFLD and the risk of early-onset endometrial cancer.

We present novel evidence that NAFLD may be an independent risk factor for early-onset endometrial cancer. Importantly, NAFLD is a modifiable condition that can be improved through increased physical activity, reduced fructose and cholesterol intake, and weight loss [44,45,46,47]. Given the high prevalence and rapid rise in NAFLD incidence, our findings support the implementation of population-based prevention strategies aimed at reducing the future burden of early-onset endometrial cancer.

The potential biological mechanisms underlying the association between NAFLD and early-onset endometrial cancer risk may be explained as follows. In NAFLD, the hepatic production and systemic release of multiple pro-oxidative mediators and proinflammatory cytokines may contribute to endometrial carcinogenesis by promoting cell proliferation, inhibiting apoptosis, inducing angiogenesis, and generating free radicals that damage DNA [20,21]. Additionally, chronic liver diseases, including NAFLD, are linked to endocrine dysregulation, characterized by systemic insulin resistance, hyperinsulinemia, elevated growth hormone levels, and hyperestrogenism [22]. Hyperestrogenism, resulting from impaired hepatic clearance of estrogen, can lead to endometrial hyperplasia and subsequent endometrial cancer development [48]. NAFLD has been implicated not only in disrupted hepatic estrogen metabolism but also in the alteration of estrogen signaling pathways within endometrial tissues [49]. Recent evidence indicates that metabolic disturbances associated with NAFLD may affect the expression and activity of estrogen receptors in the endometrium, thereby potentially enhancing estrogen-mediated proliferative signaling [50]. This mechanism may help explain the elevated risk of early-onset endometrial cancer observed in women with NAFLD. Furthermore, NAFLD-associated gut dysbiosis may promote immune dysfunction, chronic inflammation, and dysregulation of circulating estrogen levels, further facilitating endometrial carcinogenesis. [51,52,53,54] Interestingly, in our study, even individuals with normal BMI who had NAFLD (i.e., lean NAFLD) exhibited an elevated risk of early-onset endometrial cancer. Several factors are known to contribute to the etiology of lean NAFLD, including genetic predisposition, altered gut microbiota, and insulin resistance [55]. In particular, dietary patterns commonly observed in lean individuals—such as high fructose intake—may further modulate the composition of the gut microbiota and impose a glycemic burden on the liver, thereby potentially contributing to hepatic fat accumulation [56].

This study has several strengths. First, this nationwide cohort study utilized data from a large sample of more than 2.3 million women aged 20–39 years, with a follow-up period of up to 10 years. This extensive dataset enabled us to identify NAFLD as a potential modifiable risk factor for early-onset endometrial cancer. Second, the analysis incorporated blood test results, anthropometric measurements, lifestyle factors, and comprehensive medical records from a national database. The use of the KNHIS database enabled accurate tracking of the clinical course of participants following cohort entry. Third, analyses were conducted while adjusting for potential confounders, including obesity, alcohol consumption, smoking status, physical activity, diabetes, pelvic inflammatory disease, and polycystic ovary syndrome, thereby enhancing the robustness of our findings.

This study also has several limitations. First, NAFLD was not diagnosed using liver biopsy or imaging modalities. Instead, the fatty liver index employed in this study is a validated and reliable biomarker for NAFLD, demonstrating acceptable accuracy in estimating hepatic steatosis. However, the fatty liver index may have suboptimal specificity and sensitivity for detecting early or mild NAFLD compared to imaging-based diagnostic approaches, such as magnetic resonance imaging. Second, we were unable to assess participants’ family history of cancer, dietary patterns, reproductive history, or use of hormone therapy due to the unavailability of such data in the KNHIS, despite their known relevance to endometrial cancer risk. Third, information on endometrial cancer stage and histological subtypes was not available. Our study may have included a small proportion of sarcoma cases; however, more than 95% of uterine cancers are endometrial carcinomas, and uterine sarcomas are rare tumors that account for approximately 3% of all uterine cancers [57]. In addition, more than 80% of early-onset endometrial cancer cases are endometrioid adenocarcinomas. The lack of histological subtype information in our dataset limits the ability to investigate subtype-specific pathophysiological mechanisms. Further studies should investigate whether NAFLD is differentially associated with specific histological subtypes of endometrial cancer [5,8]. Finally, as our study population was limited to East Asian women, the findings may not be generalizable to other racial or ethnic groups. This highlights the need for validation studies in more diverse populations.

## 5. Conclusions

In conclusion, this study provides novel evidence that NAFLD is independently associated with an increased risk of early-onset endometrial cancer, irrespective of obesity status. Furthermore, young women with both NAFLD and obesity exhibited a synergistically elevated risk. These findings underscore the importance of early identification and targeted management of NAFLD as a potential strategy to mitigate the growing burden of early-onset endometrial cancer and reduce premature morbidity and mortality in future generations. Incorporating simple, noninvasive tools such as the fatty liver index into routine clinical assessments may enhance the identification of individuals at elevated risk. Given the modifiable nature of NAFLD, targeted public health strategies—including early screening and lifestyle interventions in reproductive-aged women—could contribute to lowering the incidence of early-onset endometrial cancer.

## Figures and Tables

**Table 1:** Baseline characteristics of the study population according to early-onset endometrial cancer status.

	Early-Onset Endometrial Cancer		*p* Value
	No	Yes	
	(*n* = 2,310,660)	(*n* = 1289)	
Age (years), mean ± SD	29.7 ± 5.2	32.3 ± 4.9	<0.001
Age groups (years), n (%)			<0.001
<30	1,200,326 (52.0)	408 (31.7)	
≥30	1,110,334 (48.1)	881 (68.4)	
BMI (kg/m^2^), mean ± SD	21.3 ± 3.2	24.3 ± 5.6	<0.001
BMI category (kg/m^2^)			<0.001
<18.5	343,329 (14.9)	126 (9.8)	
18.5–22.9	1,427,240 (61.8)	524 (40.7)	
23–24.9	268,752 (11.6)	168 (13.0)	
25–29.9	218,154 (9.4)	269 (20.9)	
≥30	53,185 (2.3)	202 (15.7)	
Waist circumference (cm), mean ± SD	70.6 ± 8.4	74.5 ± 10.7	<0.001
Systolic BP (mmHg), mean ± SD	111.2 ± 11.5	115.6 ± 13.9	<0.001
Diastolic BP (mmHg), mean ± SD	69.8 ± 8.5	72.8 ± 10.2	<0.001
Smoking status, n (%)			0.93
Never	2,108,107 (91.2)	1174 (91.1)	
Former	80,346 (3.5)	44 (3.4)	
Current	122,207 (5.3)	71 (5.5)	
Alcohol consumption ^a^, n (%)			<0.001
None	1,282,782 (55.5)	786 (61.0)	
Light to moderate	1,027,878 (44.5)	503 (39.0)	
Regular exercise, n (%)	219,329 (9.5)	140 (10.9)	0.09
Laboratory findings, mean ± SD			
Fasting glucose (mg/dL)	88.2 ± 13.1	92.4 ± 21.9	<0.001
HDL cholesterol (mg/dL)	63.3 ± 27.4	59.9 ± 31.5	<0.001
LDL cholesterol (mg/dL)	112.7 ± 306.8	118.9 ± 292.5	0.46
Triglycerides ^b^ (mg/dL)	71.6 (71.5–71.6)	89.0 (86.4–91.8)	<0.001
eGFR (mL/min/1.73 m^2^)	98.2 ± 41.3	97.1 ± 50.2	0.34
Low-income status, n (%)	501,671 (21.7)	284 (22.0)	0.78
Comorbidities, n (%)			
Diabetes	20,941 (0.9)	49 (3.8)	<0.001
Hypertension	52,711 (2.3)	99 (7.7)	<0.001
Dyslipidemia	85,084 (3.7)	103 (8.0)	<0.001
Chronic kidney disease	63,638 (2.8)	46 (3.6)	0.07
Obesity	271,339 (11.7)	471 (36.5)	<0.001

Early-onset endometrial cancer was defined as endometrial cancer diagnosed between the ages of 20 and 49 years. Data for continuous variables are presented as means ± standard deviations or geometric means (95% confidence intervals). Data for categorical variables are reported as numbers (%). ^a^ Light to moderate: <20 g alcohol/day; individuals who consumed heavy alcohol at baseline were excluded. ^b^ Geometric mean (95% CI). BMI, body mass index; BP, blood pressure; CI, confidence interval; eGFR, estimated glomerular filtration rate; HDL, high-density lipoprotein; LDL, low-density lipoprotein; SD, standard deviation.

**Table 2:** Association between NAFLD and the risk of early-onset endometrial cancer.

NAFLD	n	Event, n	Person-Years ^a^	IR ^b^	HR (95% CI)		
					Model 1	Model 2	Model 3
No	2,179,497	941	15,910,429	0.59	1 [Reference]	1 [Reference]	1 [Reference]
Yes	132,452	348	952,646	3.65	6.24 (5.51–7.05)	5.42 (4.79–6.14)	3.19 (2.76–3.67)
Moderate	92,236	155	666,101	2.33	3.96 (3.34–4.70)	3.40 (2.86–4.03)	2.38 (1.99–2.85)
Severe	40,216	193	286,545	6.74	11.57 (9.91–13.50)	10.32 (8.84–12.06)	5.39 (4.44–6.53)

^a^ Person-years represent the total accumulated follow-up time contributed by individuals within each group. ^b^ Incidence rate per 100,000 person-years. Model 1: Non-adjusted. Model 2: Adjusted for age. Model 3: Adjusted for age, smoking status, alcohol consumption, physical activity, income status, obesity, diabetes, pelvic inflammatory disease, and polycystic ovary syndrome. CI, confidence interval; HR, hazard ratio; IR, incidence rate; NAFLD, non-alcoholic fatty liver disease.

## Data Availability

The data underlying this study are derived from the National Health Insurance Service (NHIS) database of the Republic of Korea. Restrictions apply to the availability of these data, which were used under **authorization** for the current study and are not publicly available. Data may be obtained from the NHIS for researchers who meet the criteria for access to confidential data (https://nhiss.nhis.or.kr, accessed on 14 April 2025).
